# Early Maladaptive Schemas, Emotion Regulation, Stress, Social Support, and Lifestyle Factors as Predictors of Eating Behaviors and Diet Quality: Evidence from a Large Community Sample

**DOI:** 10.3390/nu17203188

**Published:** 2025-10-10

**Authors:** Małgorzata Obara-Gołębiowska

**Affiliations:** Department of Clinical, Developmental and Educational Psychology, University of Warmia and Mazury, 10-719 Olsztyn, Poland; m.obara-golebiowska@uwm.edu.pl

**Keywords:** early maladaptive schemas, emotion regulation, perceived stress, social support, eating behavior, diet quality, physical activity, obesity, health education and health promotion

## Abstract

Background: Psychological vulnerabilities, including early maladaptive schemas (EMSs), emotion regulation difficulties, perceived stress, and limited social support, are increasingly recognized as drivers of maladaptive eating and obesity. These findings underscore the need for health education and health promotion strategies that address psychological determinants of eating behavior. However, few studies integrate these psychological mechanisms with dietary and lifestyle indicators in both community and medical populations. Methods: A total of 1500 adults (aged 18–65 years; 53% women) recruited from community and medical settings participated in the study. Data were collected between January 2018 and February 2025 using standardized paper-based questionnaires. Participants completed validated measures of EMSs (YSQ-S3), emotion regulation (DERS), stress (PSS-10), social support (MSPSS), eating-related behaviors (QERB), diet (FFQ-6; Unhealthy Diet Index [UDI]), and physical activity (IPAQ-SF). Anthropometric indices included body mass index (BMI) and waist circumference (WC) as an indicator of central adiposity. Analyses involved multivariate regression, mediation, and moderation models. Results: EMSs were associated with emotional overeating and higher UDI scores. Difficulties in emotion regulation mediated the EMS–eating relationship (*β*_indirect = 0.27, *p* < 0.001). Perceived stress amplified, while social support attenuated, the association between EMSs and emotion regulation difficulties. UDI was inversely related to physical activity (*β* = −0.14, *p* < 0.01) and positively to sedentary time (*β* = 0.12, *p* < 0.01). Both BMI and WC were higher among participants reporting greater stress, emotion dysregulation, and unhealthy eating. All effects remained robust after adjustment for age, gender, and BMI. Conclusions: Early maladaptive schemas and emotion regulation difficulties contribute to unhealthy dietary patterns and central adiposity, with stress and social support acting as contextual moderators. Integrating psychological assessment with validated dietary and lifestyle measures provides a comprehensive framework for obesity prevention and schema-informed interventions. From a lifespan perspective (18–65 years), these findings highlight the need for multidomain strategies targeting cognitive–emotional and behavioral mechanisms of weight regulation.

## 1. Introduction

### 1.1. Background and Significance

Obesity has reached epidemic proportions worldwide, creating one of the most urgent public health challenges with profound physical, psychological, and socioeconomic consequences. Although diet composition and lifestyle patterns are fundamental to weight management, psychological determinants are increasingly recognized as critical factors shaping eating behaviors and long-term weight outcomes [1]. Emotional and cognitive vulnerabilities—particularly early maladaptive schemas (EMSs), difficulties in emotion regulation, high perceived stress, and low social support—have been consistently associated with disordered eating and elevated body mass index (BMI) [2,3,4]. Understanding obesity through an integrative psychological–behavioral lens is therefore essential for advancing prevention and intervention strategies.

### 1.2. Early Maladaptive Schemas and Eating Behavior

Early maladaptive schemas (EMSs) are enduring cognitive–emotional patterns that originate when core emotional needs in childhood are inadequately fulfilled [5,6]. They shape how individuals perceive themselves and others, influencing affect regulation and behavioral responses. Maladaptive schemas often manifest in adulthood through dysfunctional coping strategies, including emotional overeating and other dysregulated eating behaviors [7,8]. Empirical studies demonstrate robust associations between EMSs and diverse eating patterns such as emotional and habitual overeating as well as dietary restraint [9,10]. From a schema-based perspective, early experiences of rejection, deprivation, or impaired self-control may predispose individuals to maladaptive eating patterns later in life [11]. Systematic reviews confirm that people with overweight and obesity typically exhibit stronger endorsement of maladaptive schemas compared with normal-weight peers, suggesting that cognitive–emotional vulnerabilities play a crucial role in weight gain and maintenance [4,12]. Moreover, EMSs are often intertwined with deficits in emotion regulation, amplifying maladaptive coping tendencies and health risks [2,4].

### 1.3. Emotion Regulation and Perceived Stress

Emotion regulation encompasses the processes through which individuals monitor, evaluate, and modify their emotional experiences [13]. When these regulatory mechanisms are impaired—particularly in areas such as impulse control and emotional clarity—individuals are more likely to engage in maladaptive eating patterns and experience weight-related difficulties [7]. Research suggests that emotion regulation difficulties frequently mediate the link between EMSs and disordered eating behaviors [4,8]. 

Perceived stress represents another psychological vulnerability that influences eating habits. Large-scale studies indicate that individuals reporting higher stress levels tend to consume more ultra-processed foods, exhibit poorer diet quality, and engage more frequently in emotional eating [3,14,15]. Stress appears to exacerbate schema-driven vulnerabilities by weakening self-regulatory capacities, which in turn promotes overeating and unhealthy food choices [14,16]. Longitudinal evidence further shows that chronic stress predicts not only weight gain but also the persistence of maladaptive eating behaviors over time [17].

### 1.4. Social Support as a Protective Factor

In contrast, perceived social support functions as a key protective resource that mitigates psychological distress and promotes healthier coping. Socially supportive environments—particularly family and peer networks—can reduce the likelihood of emotional eating and improve dietary self-regulation during stressful situations [18,19]. Moreover, individuals embedded in supportive social contexts tend to report greater consumption of fruits and vegetables and better overall diet quality [20]. Thus, social support may buffer the effects of EMSs and stress on eating behavior, though these moderating mechanisms remain insufficiently studied in large community samples.

### 1.5. Diet Quality and Objective Dietary Measures

Much of the psychological research on eating behavior has relied on self-reported assessments of subjective tendencies, such as emotional or habitual overeating [21]. Although informative, these measures do not directly quantify actual dietary intake. Nutritional epidemiology underscores the importance of validated dietary assessment instruments—such as food frequency questionnaires (FFQs)—to capture real-world consumption patterns [22]. In Poland, the FFQ-6 is a standardized and validated tool used to evaluate both healthy and unhealthy dietary behaviors, including the calculation of the Unhealthy Diet Index (UDI) [23]. Higher UDI scores are indicative of poorer diet quality and are associated with obesity and related health risks [1].

Recent studies further reveal that frequent consumption of ultra-processed foods is linked not only to weight gain but also to psychological distress and emotional dysregulation [15,24]. This highlights the necessity of integrating psychological characteristics with objective measures of diet quality to comprehensively understand eating behavior.

### 1.6. Role of Physical Activity

Physical activity constitutes another lifestyle domain intimately connected with dietary and emotional functioning. The International Physical Activity Questionnaire (IPAQ) provides a reliable and widely validated tool for assessing both total activity levels and sedentary time across populations [25,26]. Evidence consistently demonstrates that physical inactivity and prolonged sitting often co-occur with unhealthy dietary patterns, magnifying metabolic and psychological risks [25]. Despite its relevance, physical activity is rarely examined in conjunction with EMSs, emotion regulation, and diet quality within a single conceptual framework. Established methodological standards for IPAQ data processing further support its integration into multidisciplinary health research [26].

### 1.7. Rationale and Aims of the Present Study

Cumulative findings emphasize that EMSs, emotion regulation difficulties, stress, and social support jointly shape eating behavior and weight outcomes. Nevertheless, few studies have examined these psychological constructs alongside objective dietary indices (e.g., UDI) and physical activity measures. Most prior research has focused narrowly on subjective eating tendencies, limiting insight into broader lifestyle mechanisms underlying obesity.

The present study addresses this gap by analyzing how early maladaptive schemas and difficulties in emotion regulation contribute to maladaptive eating and diet-related risk, while also testing whether stress and social support moderate these associations. Additionally, physical activity and sedentary behavior were examined as complementary lifestyle indicators. Through this integrative perspective, the study aims to advance understanding of psychological and behavioral mechanisms contributing to obesity and to inform schema-based preventive strategies. This work forms part of a broader research program on psychological and lifestyle determinants of body weight, which has previously explored group-level and genetic correlates in related analyses [27,28].

## 2. Materials and Methods

### 2.1. Participants

This study employed a stratified purposive sampling method to ensure balanced representation across body mass index (BMI) categories (normal weight vs. overweight/obese), age groups (younger vs. older adults), and gender. This approach was chosen to control for key demographic and psychological variables while maintaining diversity in the study population.

Participants were recruited in Olsztyn and the surrounding areas in northeastern Poland through local universities, healthcare facilities (including an obesity treatment clinic), and one sports center. Data collection was conducted between January 2018 and February 2025 using the paper-and-pencil format. The extended recruitment period was required due to the large target sample size and the comprehensive nature of the study protocol, which included in-person anthropometric assessments and several validated psychometric instruments. To minimize potential bias related to pandemic-associated emotional and behavioral disturbances, no data were collected during the strict national COVID-19 lockdowns in 2020.

**Figure 1:**
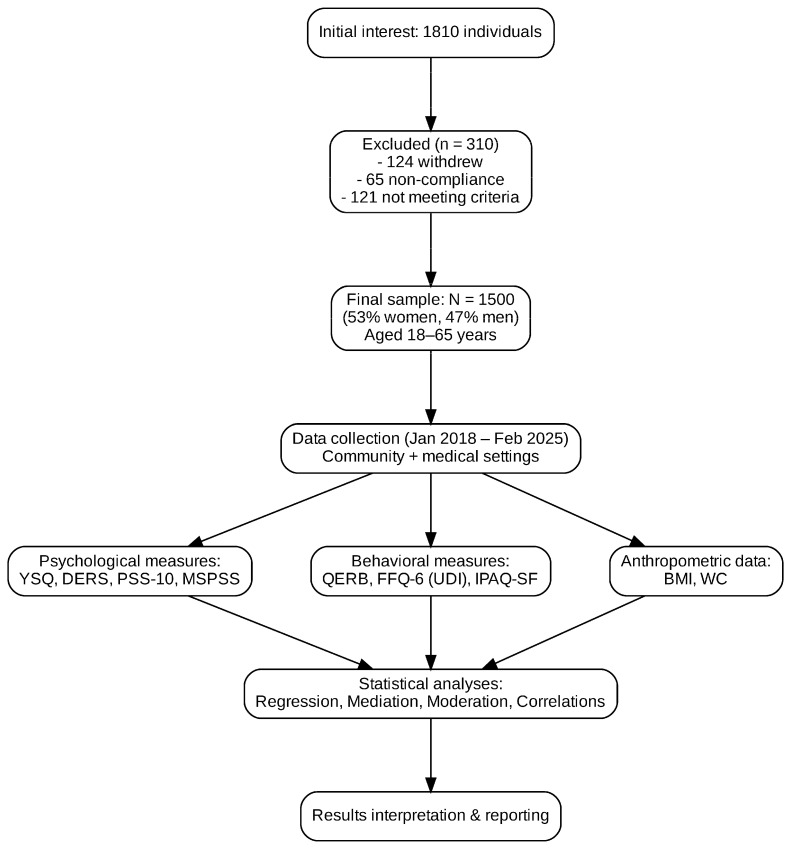
Flowchart illustrating participant recruitment, exclusion process, and data collection stages. The diagram presents the total number of individuals initially screened, reasons for exclusion (withdrawal, non-compliance, and unmet inclusion criteria), and the final analytic sample (*N* = 1500) stratified by BMI category, gender, and age group.

An initial pool of 1810 individuals expressed interest in participation. Of these, 310 were excluded: 124 withdrew before completing the protocol, 65 were excluded for non-compliance, and 121 did not meet inclusion criteria. The final analytic sample comprised *N* = 1500 adults (53.0% women), with a mean age of 36.8 years (*SD* = 11.9, range = 18–65 years), stratified according to BMI status, gender, and age group (Figure 1). Sociodemographic characteristics of the participants are presented in Table 1.

The same dataset was used in a recently published study (Obara-Gołębiowska, 2025) [27], which examined group differences in early maladaptive schemas, emotion regulation, and eating behaviors. However, the present article addresses a distinct set of research questions, expands the range of variables (including dietary intake indices, perceived stress, social support, and physical activity), and tests novel hypotheses regarding mediation and moderation pathways.

In addition to overall descriptive statistics, sex differences were examined across all psychological, dietary, and lifestyle variables. As shown in Table 2, women reported significantly higher levels of emotional and habitual overeating, dietary restraint, emotion regulation difficulties, perceived stress, and perceived social support, whereas men showed higher BMI, waist circumference, and slightly longer sitting time (all *p* < 0.05). These results are consistent with established gender-related psychological and lifestyle patterns.

### 2.2. Inclusion and Exclusion Criteria

Inclusion criteria were: (a) age between 18 and 65 years, (b) BMI classified as normal weight (18.5–24.9 kg/m^2^) or overweight/obese (≥25.0 kg/m^2^) according to WHO guidelines, and (c) ability to understand study procedures and provide written informed consent.

Exclusion criteria included: pregnancy or lactation; legal incapacity or inability to provide informed consent; severe psychiatric disorders (e.g., schizophrenia, bipolar disorder); current psychiatric treatment for severe conditions or a history of psychiatric hospitalization; intellectual disability or cognitive impairment precluding completion of self-report questionnaires; self-reported alcohol or drug dependence; and medical conditions known to significantly affect weight regulation or metabolic functioning (e.g., type 1 diabetes, active endocrine disorders).

### 2.3. Screening for Psychiatric and Medical Conditions

Before enrollment, participants completed a brief structured medical history questionnaire. This tool screened for previous psychiatric diagnoses, psychiatric hospitalizations, ongoing psychiatric treatment, and substance use. Individuals reporting severe psychiatric conditions or substance use disorders were excluded from the primary analyses.

### 2.4. Anthropometric Assessment

Anthropometric measurements were obtained under standardized conditions. Body weight was measured with a calibrated digital scale and height with a stadiometer. BMI was calculated as weight (kg) divided by height squared (m^2^). Waist circumference (WC) was measured at the midpoint between the iliac crest and the lower rib margin. Based on BMI, participants were classified as normal weight (18.5–24.9 kg/m^2^) or overweight/obese (≥25.0 kg/m^2^).

### 2.5. Measures

Early Maladaptive Schemas. The Polish adaptation/validation of the Young Schema Questionnaire—Short Form 3 (YSQ-S3) was used to assess EMSs (90 items, 18 schemas, 6-point Likert) [5,29]. The instrument consists of 90 items assessing 18 cognitive–emotional patterns, rated on a 6-point Likert scale (1 = “Completely untrue of me” to 6 = “Describes me perfectly”). A total score (YSQ_Total) was computed, with higher scores reflecting greater intensity of maladaptive schemas. The Polish version used in this study demonstrated good psychometric validity and reliability. Internal consistency in the present study: Cronbach’s α = 0.923.

Difficulties in Emotion Regulation. Emotion regulation was measured with the Difficulties in Emotion Regulation Scale (DERS) [30,31], comprising 36 items across six domains (nonacceptance of emotions, goals, impulse, awareness, strategies, clarity). Items were rated on a 5-point Likert scale (1 = “Almost never” to 5 = “Almost always”), with higher scores indicating greater emotion regulation difficulties. The Polish adaptation and validation by Dragan [31] was used, showing satisfactory reliability. Cronbach’s α = 0.875.

Eating-Related Behaviors. Maladaptive eating patterns were assessed with the Questionnaire of Eating-Related Behaviors (QERB) [32], developed and validated in Poland. The tool includes three subscales: Emotional Overeating (EO), Habitual Overeating (HO), and Dietary Restraint (DR). Items are rated from 0 (“Never”) to 10 (“Always”), with higher scores indicating greater intensity of the specific eating behavior. In the present study, the internal consistency of the overall scale was excellent (Cronbach’s α = 0.933).

Perceived Stress. The Perceived Stress Scale (PSS-10) [33,34] was used to assess the subjective intensity of stress during the past month. It consists of 10 items rated on a 5-point scale (0 = “Never” to 4 = “Very often”), with higher scores indicating greater perceived stress. The Polish adaptation and validation by Ogińska-Bulik and Juczyński [34] was employed, showing strong psychometric properties. Cronbach’s α = 0.846.

Perceived Social Support. Perceived social support was measured with the Multidimensional Scale of Perceived Social Support (MSPSS) [35,36], assessing support from family, friends, and significant others. The instrument includes 12 items rated on a 7-point Likert scale (1 = “Very strongly disagree” to 7 = “Very strongly agree”). A composite score (MSPSS_Total) was calculated to reflect global perceived support. The Polish validated version by Buszman and Przybyła-Basista was used [36]. Cronbach’s α = 0.902.

Dietary Intake. A shortened version of the Food Frequency Questionnaire (FFQ-6) [23] was administered to assess dietary intake. Six food categories were included: sweets, fast food, sugary drinks, fruits and vegetables, whole grains, and processed meats. Responses were provided on a 6-point Likert scale (1 = “Never or almost never” to 6 = “Daily or almost daily”). An Unhealthy Diet Index (UDI) was computed as the mean score of sweets, fast food, sugary drinks, and processed meats, with higher values reflecting greater consumption of calorie-dense, low-nutrient foods. The Polish validated FFQ-6 version was applied [23]. Cronbach’s α = 0.821.

Physical Activity and Sedentary Behavior. The Polish version of the International Physical Activity Questionnaire (IPAQ-SF) [24,37] was used to assess physical activity across work, transport, household, and leisure domains. Total physical activity was expressed in MET-min/week. Sedentary behavior was assessed by the mean number of sitting hours per day. Cronbach’s α was not applicable, as IPAQ is a structured activity recall.

### 2.6. Ethical Considerations

The study protocol was approved by the Ethics Committee for Scientific Research at the University of Warmia and Mazury in Olsztyn, Poland (approval no. 1/2017). All participants provided written informed consent before participation. The study was conducted in accordance with the Declaration of Helsinki.

### 2.7. Statistical Analysis

All statistical analyses were conducted in Python 3.11 using the following libraries: pandas and NumPy for data management, SciPy for preliminary tests, statsmodels for regression and MANOVA, and semopy for structural equation modeling. Prior to inferential analyses, assumptions of normality and homogeneity of variance were assessed with the Shapiro–Wilk and Levene’s tests. Descriptive statistics (*M*, *SD*, range) were computed for all variables.

Pearson correlations were calculated to examine associations among EMSs, emotion regulation, perceived stress, social support, eating behaviors, UDI, and physical activity. Multivariate analyses of variance (MANOVA) were performed to test group differences across BMI, gender, and age. Multiple regression models were estimated to identify predictors of eating behaviors and dietary risk.

Mediation models tested whether emotion regulation mediated the relationship between EMSs and (a) emotional overeating and (b) UDI. Moderation models assessed whether perceived stress and social support influenced the pathway from EMSs to emotion regulation. Bootstrapping procedures (5000 resamples) were used to calculate bias-corrected 95% confidence intervals. Effect sizes were reported as partial *η*^2^ for MANOVA, standardized β coefficients for regression, and Cohen’s d for pairwise comparisons. Statistical significance was set at α = 0.05 (two-tailed).

## 3. Results

### 3.1. Preliminary Statistical Analyses

A priori power analysis was conducted to evaluate the sensitivity of the study sample (*N* = 1500) for detecting effects in multiple regression models with up to six predictors. Assuming a small-to-medium effect size (*f*^2^ = 0.05), α = 0.05, and power = 0.95, the minimum required sample size was estimated at *N* = 263. The actual sample of *N* = 1500 therefore substantially exceeded this threshold, ensuring adequate power to detect even small effects and providing descriptive statistics, including means, standard deviations, minimum and maximum values, skewness, and kurtosis, were calculated for all study variables (Table 2). These indices confirmed sufficient variability across psychological, dietary, and behavioral measures. In addition, waist circumference (WC) was included as an anthropometric indicator of central adiposity. Descriptive statistics for WC (*M* = 90.22 cm, *SD* = 14.74, range = 60–131) are presented in Table 3. The distribution was approximately normal (skewness = 0.12, kurtosis = −0.56), and WC was strongly correlated with BMI (*r* = 0.79, *p* < 0.001). Moderate-to-strong associations were also observed between WC and eating-related variables (*r* = 0.53–0.67) as well as emotion regulation difficulties (*r* = 0.42, *p* < 0.001), while higher perceived stress and lower physical activity were related to greater WC. Full correlation coefficients of WC with psychological, dietary, and lifestyle variables are provided in Supplementary Table S1. In addition to the overall descriptive statistics, sex differences were examined across all psychological, dietary, and lifestyle variables. As shown in Table 2, women reported significantly higher levels of emotional and habitual overeating, dietary restraint, emotion regulation difficulties, perceived stress, and perceived social support, whereas men showed higher BMI, waist circumference, and slightly longer sitting time (all *p* < 0.05). These results are consistent with established gender-related psychological and lifestyle patterns.

Normality of distributions was evaluated using the Shapiro–Wilk test. Given the large sample size, most variables showed significant deviations from normality (*p* < 0.05), which is common in large datasets. However, skewness and kurtosis values for all continuous variables were within acceptable thresholds (|skewness| < 2, |kurtosis| < 7), supporting the use of parametric procedures such as Pearson correlations, regression analyses, and structural equation modeling. Therefore, no data transformations were applied. Pearson correlations among all study variables are provided in Supplementary Table S2.

Internal consistency of all psychometric scales was satisfactory, with Cronbach’s alpha values exceeding 0.84 across instruments. Outlier detection based on z-scores (>|3.0|) did not reveal any extreme values requiring exclusion. Additional detailed results, including extended regression models, supplementary mediation analyses, and sensitivity tests, are available in the Supplementary Materials (Tables S3–S5).

### 3.2. Mediation and Moderation Analyses

Mediation models tested whether difficulties in emotion regulation (DERS) explained the relationship between early maladaptive schemas (YSQ) and eating-related outcomes (see Table 4). Across all models, DERS significantly mediated the association between EMSs and emotional overeating (EO) as well as unhealthy diet (UDI). Indirect effects remained robust after controlling for age, gender, and BMI (see also Supplementary Table S4 for extended models including both outcomes).

Moderation analyses further demonstrated that perceived stress (PSS-10) strengthened the relationship between EMSs and DERS. Specifically, individuals with higher levels of perceived stress exhibited stronger links between maladaptive schemas and emotion regulation difficulties. In contrast, social support (MSPSS) attenuated this pathway, serving as a protective factor that reduced the impact of EMSs on DERS. Neither PSS nor MSPSS moderated the direct paths between DERS and eating-related outcomes, suggesting that their primary influence operates on the schema–regulation link rather than on eating behavior per se. Additional regression models predicting specific eating behaviors are presented in Supplementary Table S3, while direct associations of perceived stress with eating behaviors are provided in Supplementary Table S6. These findings are consistent with analyses previously conducted on the same dataset [27], which highlighted EMSs and DERS as core psychological vulnerabilities across BMI, gender, and age. The current results extend this by showing that stress and social support function as contextual amplifiers or buffers of schema-related regulation difficulties.

Additional analyses of perceived stress, including its direct associations with eating behaviors and adjusted regression models, are presented in Supplementary Table S6.

### 3.3. Associations with Physical Activity

Analyses incorporating lifestyle outcomes revealed that higher UDI scores were significantly associated with lower total physical activity (*r* = −0.21, *p* < 0.001) and greater weekly sitting time (*r* = 0.18, *p* < 0.001). Emotional overeating was also negatively correlated with physical activity (*r* = −0.15, *p* < 0.01).

Regression models (Table 5) confirmed that UDI was predicted not only by psychological variables (YSQ, DERS, stress, social support) but also by physical activity indicators. Specifically, higher sitting time independently predicted higher UDI (*β* = 0.12, *p* < 0.01), whereas higher total MET-minutes predicted lower UDI (*β* = −0.14, *p* < 0.01). Sensitivity analyses across BMI subgroups are presented in Supplementary Table S5, indicating that these associations were consistent across groups, with slightly stronger effects in overweight/obese participants.

## 4. Discussion

The present study explored the interrelations among early maladaptive schemas (EMSs), emotion regulation, perceived stress, social support, dietary intake, and physical activity in a large community sample of adults. The findings confirmed that psychological mechanisms are closely intertwined with eating behaviors and weight-related risk factors. In particular, EMSs and emotion regulation difficulties emerged as prominent predictors of emotional and habitual overeating, consistent with schema theory and prior empirical research [2,4,7,8,9,10,11,12]. These results reinforce the notion that obesity should not be viewed solely as a metabolic disorder but also as a condition shaped by enduring cognitive–emotional structures that influence self-regulation and behavior [5,6,11]. Furthermore, consistent gender-related patterns were observed, with women reporting greater emotional and stress-related vulnerability and men showing higher anthropometric indicators and sedentary behavior, mirroring well-established differences in psychological and lifestyle domains.

Comparable findings have been reported in diverse cultural contexts. For example, Murayama and Ohya [38] observed that Japanese women with stronger tendencies toward rumination and suppression exhibited more abnormal eating behaviors, including overeating and bulimic symptoms. Similarly, Luadlai et al. [39] found that both positive and negative affect were linked to overeating in Thai adults, with emotion regulation difficulties mediating this relationship.

Consistently with prior studies [8,13], emotion regulation difficulties were strongly associated with maladaptive eating. Deficits in impulse control and limited access to adaptive strategies likely intensify susceptibility to overeating, especially in the presence of stress or negative affect. The observed associations support previous evidence indicating that emotion regulation mediates the relationship between EMSs and disordered eating [4,7], highlighting the importance of strengthening emotional competencies in interventions targeting obesity and overeating.

This association appears to generalize cross-culturally. Studies in Italy and Turkey have demonstrated that emotion regulation difficulties predict unhealthy eating and higher BMI across different populations [40,41]. Favieri et al. [40], in a systematic review, confirmed that deficits in adaptive strategies such as reappraisal consistently increase the risk of emotional overeating among adolescents and adults. Ackermans et al. [42] further showed that individuals with more adaptive cognitive regulation consumed less food under negative emotions, emphasizing the behavioral consequences of emotion regulation quality. Arslan et al. [41] also reported that higher difficulties in emotion regulation predicted unhealthy eating attitudes and higher BMI among Turkish university students.

Perceived stress emerged as another robust predictor of unhealthy dietary behavior. Participants reporting higher stress levels demonstrated greater consumption of ultra-processed foods and poorer overall diet quality, mirroring meta-analytic findings linking stress to unhealthy dietary choices and emotional eating [3,14,15]. Stress is known to impair self-regulation and amplify schema-driven vulnerabilities [16,17]. Importantly, in the present data, stress-related dietary risk was evident not only in subjective tendencies toward overeating but also in objective indices of diet quality, such as the Unhealthy Diet Index (UDI) [23]. These converging results underscore the cumulative influence of stress on both psychological and behavioral domains of eating.

Recent international data confirm this pattern. For instance, Elhadidy et al. [43], in a large Italian sample, found that emotional overeating was closely associated with perceived stress and the frequent use of online food-delivery services, suggesting that environmental and emotional pressures jointly shape maladaptive eating.

Alongside BMI, waist circumference (WC) was analyzed as an indicator of central adiposity. The associations between WC and psychological or behavioral variables closely paralleled those found for BMI: higher emotion dysregulation, greater perceived stress, and more unhealthy eating were all linked to increased WC. This convergence suggests that cognitive–emotional and lifestyle factors contribute not only to overall adiposity but also to body-fat distribution associated with metabolic risk. Given that central adiposity is a stronger predictor of cardiometabolic complications than BMI alone, these results highlight the clinical relevance of incorporating psychological assessment into obesity prevention and management strategies.

In contrast, perceived social support was associated with healthier dietary patterns, consistent with previous research demonstrating that supportive social environments buffer distress and facilitate more adaptive eating behaviors [18,19,20]. Social support may counteract the detrimental effects of EMSs and stress by enhancing coping resources and promoting stability in health-related routines. Nevertheless, the protective function of social ties appeared partial, implying that social resources alone cannot fully offset cognitive–emotional vulnerabilities. Future research should investigate whether interventions aimed at strengthening interpersonal support can enhance the efficacy of schema-based and emotion-focused approaches.

The findings related to dietary intake further emphasize the importance of integrating objective nutritional measures into psychological research. While many prior studies have relied primarily on self-reported measures of maladaptive eating [21], the current analysis employed a validated food frequency questionnaire (FFQ-6) and confirmed that higher UDI scores are associated with obesity-related risk [22,23]. These results align with accumulating evidence that frequent consumption of ultra-processed foods is linked not only to weight gain but also to emotional dysregulation and psychological distress [14,15,16,24]. Collectively, these findings highlight the necessity of combining psychological constructs with validated nutritional metrics to obtain a comprehensive understanding of diet-related risk.

Physical activity, measured using the International Physical Activity Questionnaire (IPAQ), played a complementary role. Low physical activity and extended sedentary time coexisted with maladaptive eating patterns, illustrating the clustering of unhealthy lifestyle behaviors [25,26]. These results are consistent with systematic reviews confirming the validity of IPAQ-based indices and their utility in linking psychological and physiological health correlates [25]. Incorporating physical-activity indicators into schema-focused models provides a more integrated perspective on obesity risk and opens new avenues for multidomain interventions.

Taken together, the present findings expand upon earlier research by concurrently analyzing EMSs, emotion regulation, stress, social support, dietary intake, and physical activity within a single conceptual framework. This comprehensive approach supports the conceptualization of obesity as a multifaceted biopsychosocial phenomenon [1,27]. Furthermore, the current results complement previous analyses from the same research program, which examined group-level differences in EMSs and eating behaviors across BMI, gender, and age [27], and build upon earlier pilot findings indicating that psychological and lifestyle factors could be at least as relevant as genetic variation in the FTO gene for understanding body-mass differences [28].

Collectively, evidence from different world regions—including Europe, Asia, and the Middle East—underscores the universality of the observed cognitive–emotional mechanisms, suggesting that emotion regulation and stress processes may represent cross-cultural determinants of eating behavior and weight regulation [38,39,40,41,42,43].

### 4.1. Practical Implications

The findings suggest that effective interventions for overweight and obesity should target not only diet and exercise but also underlying cognitive–emotional mechanisms. Integrating schema-therapy principles, emotion-regulation training, and stress-management techniques into lifestyle interventions may help address the root causes of maladaptive eating [11,29,30,31]. Additionally, strengthening social-support networks could enhance resilience against stress-related overeating and foster adherence to healthier eating patterns [18,19,20].

Given the converging international evidence, culturally adapted interventions may be especially beneficial, addressing both universal and culture-specific aspects of emotion regulation and eating behavior. Such approaches could increase the relevance and effectiveness of schema-informed, multidomain interventions across diverse populations.

### 4.2. Limitations and Future Directions

Several limitations should be considered when interpreting these results. First, the cross-sectional design precludes causal inference, and reliance on self-report measures may introduce reporting bias. Although most instruments captured psychological constructs, the FFQ-6 and IPAQ specifically assessed dietary intake and physical activity. Future studies should expand on these findings by examining nutrient composition and dietary subpatterns in relation to psychological factors. The current sample was drawn from a Polish population, which may limit generalizability to other cultural or socioeconomic contexts.

The inclusion of cross-cultural comparisons in future studies would further clarify the universality versus cultural specificity of schema-related mechanisms. Longitudinal and interventional research across various populations could also determine whether emotion regulation and stress responses function as consistent mediators of unhealthy eating behaviors globally.

Furthermore, integrating genetic markers such as FTO variants with psychological and lifestyle predictors could enhance the precision of future obesity research [28].

## 5. Conclusions

This study provides strong evidence that EMSs, emotion-regulation difficulties, and stress represent key psychological predictors of maladaptive eating and poor diet quality, whereas social support serves as a partial protective factor. These findings underscore the importance of addressing cognitive–emotional vulnerabilities alongside dietary and behavioral components in obesity prevention and treatment. Adopting a schema-informed, multidomain approach may improve both psychological well-being and long-term weight outcomes.

Building on these results, future research should integrate biological, psychological, and lifestyle factors and extend cross-cultural analyses to better capture the complex determinants of obesity and eating behavior worldwide.

## Figures and Tables

**Table 1:** Sociodemographic Characteristics of the Study Sample (*N* = 1500).

Variable	Categories	*n* (%)
Gender	Female/Male	795 (53.0)/705 (47.0)
Age group	Younger (18–35)/Older (36–65)	825 (55.0)/675 (45.0)
Residence	Urban/Rural	870 (58.0)/630 (42.0)
Education	Higher/Secondary	675 (45.0)/825 (55.0)

Note. Values are presented as number and percentage of participants within each category.

**Table 2:** Sex Differences in Psychological, Behavioral, and Lifestyle Variables (N = 1500).

Variable	Female *M* (*SD*)	Male *M* (*SD*)	*t*	*p*	Cohen’s *d*
QERB—Emotional Overeating	6.06 (1.68)	5.34 (1.69)	8.25	<0.001	0.43
QERB—Habitual Overeating	6.13 (1.74)	5.36 (1.69)	8.72	<0.001	0.45
QERB—Dietary Restraint	6.04 (1.68)	5.30 (1.67)	8.51	<0.001	0.44
DERS—Total	115.30 (12.36)	107.76 (12.80)	11.59	<0.001	0.60
PSS-10—Perceived Stress	23.32 (3.97)	20.63 (4.11)	12.87	<0.001	0.67
MSPSS—Perceived Social Support	65.15 (8.18)	59.33 (8.23)	13.72	<0.001	0.71
UDI—Unhealthy Diet Index	3.87 (0.40)	3.80 (0.43)	3.18	0.001	0.16
IPAQ—Total MET-min/week	5084.78 (1563.35)	4910.89 (1531.06)	2.18	0.030	0.11
IPAQ—Sitting Time (hours/day)	4.27 (0.55)	4.34 (0.53)	−2.47	0.014	−0.13
BMI (kg/m^2^)	25.49 (4.67)	26.37 (4.94)	−3.56	<0.001	−0.18
Waist Circumference (cm)	96.00 (13.08)	84.05 (13.89)	17.12	<0.001	0.89

Note. Values are presented as means and standard deviations (*M* ± *SD*). Positive *t* and *d* values indicate higher means among women; negative values indicate higher means among men.

**Table 3:** Descriptive Statistics of Study Variables (*N* = 1500).

Variable	*M*	*SD*	Min	Max	Skewness	Kurtosis
BMI (kg/m^2^)	25.92	4.82	18.54	34.97	0.26	−1.17
Waist circumference (cm)	90.22	14.74	60.00	131.10	0.12	−0.56
QERB—Emotional Overeating	7.71	1.72	2.15	9.86	0.11	−0.96
QERB—Habitual Overeating	5.76	1.76	2.08	9.83	0.10	−1.09
QERB—Dietary Restraint	5.68	1.72	2.02	9.76	0.16	−0.92
DERS—Total	111.65	13.12	77.05	147.60	−0.03	−0.41
PSS-10	22.02	4.25	10.72	33.12	−0.04	−0.43
MSPSS—Total	62.33	8.70	41.50	83.86	0.01	−0.40
YSQ—Total	71.65	8.46	52.61	94.60	0.03	−0.59
UDI—Unhealthy Diet Index	3.24	0.91	1.00	5.75	0.27	−0.65
IPAQ—Total MET-min/week	3125.48	1742.61	480.00	9640.00	0.41	−0.54
IPAQ—Sitting Time (hours/week)	39.62	11.75	10.00	75.00	0.22	−0.71

Note. BMI = Body Mass Index; WC = Waist Circumference; QERB = Questionnaire of Eating-Related Behaviors; DERS = Difficulties in Emotion Regulation Scale; PSS-10 = Perceived Stress Scale; MSPSS = Multidimensional Scale of Perceived Social Support; YSQ = Young Schema Questionnaire; UDI = Unhealthy Diet Index (calculated from FFQ-6 categories); IPAQ = International Physical Activity Questionnaire.

**Table 4:** Mediation and Moderation Analyses for Emotional Overeating and Unhealthy Diet Index.

Pathway	*β*	SE	95% CI	*p*
YSQ → DERS	0.42	0.03	[0.36, 0.48]	<0.001
DERS → Emotional Overeating	0.27	0.03	[0.22, 0.33]	<0.001
YSQ → Emotional Overeating (direct)	0.18	0.04	[0.10, 0.26]	<0.001
DERS → UDI	0.19	0.04	[0.12, 0.26]	<0.001
YSQ → UDI (direct)	0.14	0.05	[0.05, 0.23]	<0.01
Stress × YSQ → DERS	0.08	0.02	[0.04, 0.12]	<0.01
Social Support × YSQ → DERS	−0.09	0.02	[−0.13, −0.05]	<0.01

Note. *β* = standardized regression coefficient; SE = standard error; CI = confidence interval; YSQ = Young Schema Questionnaire; DERS = Difficulties in Emotion Regulation Scale; UDI = Unhealthy Diet Index.

**Table 5:** Associations between Physical Activity, Sitting Time, and Dietary Risk.

Predictor	Outcome: UDI	*β*	SE	*p*
Total MET-min/week (IPAQ)	−0.14	0.04	<0.01	
Sitting time (hours/week)	0.12	0.04	<0.01	

Note. *β* = standardized regression coefficient; SE = standard error; UDI = Unhealthy Diet Index; IPAQ = International Physical Activity Questionnaire.

## Data Availability

The data presented in this study are available on request from the corresponding author due to ethical and privacy restrictions.

## Supplementary Materials

The following supporting information can be downloaded at: https://www.mdpi.com/article/10.3390/nu17203188/s1. Table S1: Full correlation matrix of all study variables; Table S2: Regression models predicting eating behaviors; Table S3: Extended mediation models; Table S4: Sensitivity analyses for physical activity across BMI groups; Table S5: Direct effects of perceived stress on eating behaviors; Table S6: Direct Effects of Perceived Stress (PSS-10) on Eating Behaviors.
