# Supplementary material for: Early Maladaptive Schemas, Emotion Regulation, Stress, Social Support, and Lifestyle Factors as Predictors of Eating Behaviors and Diet Quality: Evidence from a Large Community Sample
Source: Nutrients. 2025 Oct 10;17(20):3188. doi: 10.3390/nu17203188 (PMC12566790; doi:10.3390/nu17203188)
Supplement: Supplementary file 1 [file nutrients-17-03188-s001.zip › nutrients-3916914-supplementary.pdf]

Table S1. Correlations of Waist Circumference with Psychological, Dietary, and Lifestyle Variables (N = 1500)

Variable	r (Pearson)	p
BMI	0.79	< 0.001
QERB – Emotional Overeating	0.62	< 0.001
QERB – Habitual Overeating	0.67	< 0.001
QERB – Dietary Restraint	0.66	< 0.001
DERS – Difficulties in Emotion Regulation	0.42	< 0.001
PSS-10 – Perceived Stress	0.39	< 0.001
MSPSS – Social Support	–0.07	n.s.
UDI – Unhealthy Diet Index	0.53	< 0.001
IPAQ – Total MET-min/week	–0.23	< 0.001
IPAQ – Sitting Time (hours/week)	0.33	< 0.001

Note. WC = Waist Circumference; BMI = Body Mass Index; QERB = Questionnaire of Eating-Related Behaviors; DERS = Difficulties in Emotion Regulation Scale; PSS-10 = Perceived Stress Scale; MSPSS = Multidimensional Scale of Perceived Social Support; UDI = Unhealthy Diet Index (derived from FFQ-6); IPAQ = International Physical Activity Questionnaire. All coefficients are Pearson's *r*.

Table S2. Full Correlation Matrix of All Study Variables (N = 1500)

Variable	BMI	YSQ_Total	DERS_Total	PSS-10	MSPSS_Total	EO	HO	DR	UDI	IPAQ_MET	Sitting
**BMI**	1	0.22***	0.19***	0.16***	-0.14***	0.21***	0.20***	0.18***	0.17***	-0.12***	0.09**
**YSQ_Total**		1	0.46***	0.32***	-0.28***	0.38***	0.36***	0.30***	0.29***	-0.18***	0.14***
**DERS_Total**			1	0.44***	-0.31***	0.41***	0.38***	0.27***	0.33***	-0.20***	0.15***
**PSS-10**				1	-0.37***	0.34***	0.32***	0.19***	0.28***	-0.15***	0.17***
**MSPSS_Total**					1	-0.26***	-0.25***	-0.18***	-0.22***	0.13***	-0.11***
**EO**						1	0.52***	0.31***	0.28***	-0.15***	0.12***
**HO**							1	0.34***	0.26***	-0.13***	0.10**
**DR**								1	0.18***	-0.09**	0.07*
**UDI**									1	-0.21***	0.18***
**IPAQ_MET**										1	-0.29***
**Sitting**											1

Note. EO = Emotional Overeating; HO = Habitual Overeating; DR = Dietary Restraint. Reported values are Pearson correlation coefficients. Significance levels: *p < 0.05, **p < 0.01, ***p < 0.001.

Table S3. Regression Models Predicting Emotional Overeating (EO), Habitual Overeating (HO), and Dietary Restraint (DR)

Predictor	EO (β)	HO (β)	DR (β)
YSQ_Total	0.21***	0.20***	0.17***
DERS_Total	0.27***	0.23***	0.15***
PSS-10	0.18***	0.16***	0.09**
MSPSS_Total	-0.14***	-0.12***	-0.07*
**R²**	0.38	0.34	0.26

Note. Multiple linear regression models with standardized coefficients. R^2 = explained variance. EO = Emotional Overeating; HO = Habitual Overeating; DR = Dietary Restraint. Significance levels: *p < 0.05, **p < 0.01, ***p < 0.001.

Table S4. Extended Mediation Models Including Both Outcomes (EO and UDI)

Pathway	Indirect Effect (β)	SE	95% CI	p
YSQ → DERS → EO	0.27	0.03	[0.22, 0.33]	<0.001
YSQ → DERS → UDI	0.19	0.04	[0.12, 0.26]	<0.001

Note. Mediation tested with 5000 bootstrap samples. EO = Emotional Overeating; UDI = Unhealthy Diet Index; YSQ = Young Schema Questionnaire; DERS = Difficulties in Emotion Regulation Scale.

Table S5. Sensitivity Analyses: Physical Activity Predicting UDI across BMI Groups

Predictor	Normal Weight (β)	Overweight/Obese (β)
IPAQ_MET	-0.12**	-0.16***
Sitting Time	0.09*	0.15***

Note. β = standardized regression coefficient. Results indicate consistent associations across BMI subgroups, with slightly stronger effects in overweight/obese participants. Significance levels: *p < 0.05, **p < 0.01, ***p < 0.001.

Table S6. Direct Effects of Perceived Stress (PSS-10) on Eating Behaviors

Panel A. Bivariate standardized regressions (predictor: PSS-10)

Outcome (dependent variable)	β (standardized)	p-value
Emotional Overeating (EO)	0.34	***
Habitual Overeating (HO)	0.32	***
Dietary Restraint (DR)	0.19	***

Panel B. Minimally adjusted standardized regressions

(predictor: PSS-10; covariates: age, gender, BMI)

Outcome (dependent variable)	β (standardized)	p-value
Emotional Overeating (EO)	0.18	***
Habitual Overeating (HO)	0.16	***
Dietary Restraint (DR)	0.09	**

Note. β = standardized regression coefficient. Stars denote significance: ** $p < 0.01$, *** $p < 0.001$. Panel A shows direct associations between PSS-10 and eating behaviors (consistent with correlations in Table S1). Panel B presents effects after adjusting for age, gender, and BMI. In full models including EMS (YSQ) and emotion regulation difficulties (DERS) (see Table S3), direct effects of PSS-10 were no longer significant. Instead, perceived stress primarily emerged as a moderator of the EMS → DERS pathway (see Table 3 in the main text).
